# Decoding patient preferences: key drivers in selecting thyroid cancer surgery- a discrete choice experiment

**DOI:** 10.3389/fendo.2026.1705391

**Published:** 2026-06-17

**Authors:** Shiwei Zhou, Yu Mao, Peng Wu, Wu Li, Hui Li, Xiaohua Song, Xiaoyong Wen, Zeyu Li, Guangji Chen, Xiaowei Peng

**Affiliations:** 1 Department of Thyroid Surgery, The Affiliated Cancer Hospital of Xiangya School of Medicine, Central South University/Hunan Cancer Hospital, Changsha, Hunan, China; 2 Department of Thyroid Surgery, the Second Xiangya Hospital, Central South University, Changsha, Hunan, China; 3 Department of Surgery, University Hospital, Central South University, Changsha, Hunan, China

**Keywords:** cosmetic concerns, discrete choice experiment, papillary thyroid carcinoma, recurrence rate, transoral endoscopic thyroidectomy vestibular approach

## Abstract

**Objective:**

With the growing application of the transoral endoscopic thyroidectomy vestibular approach (TOETVA) for papillary thyroid carcinoma (PTC), understanding patient preferences is critical to improving communication and supporting informed decision-making. This study aimed to identify key factors influencing surgical choices.

**Method:**

A discrete choice experiment was conducted with patients diagnosed with PTC who were scheduled for thyroidectomy. Participants evaluated hypothetical surgical scenarios varying in six attributes: incision location, incision size, recurrence rate, operative duration, complication rate, and cost. Preferences and willingness to pay (WTP) were analyzed using a mixed logit model.

**Results:**

A total of 271 patients were included in this study. Patients demonstrated a strong preference for surgical options with lower recurrence rates and scarless approaches. They were willing to pay an additional ¥201,409.60 to reduce the recurrence rate from 10% to 1% and ¥19,141.14 to change the surgical scar location from external to internal. The recurrence rate emerged as the most crucial non-financial factor (β = 8.18, *P* < 0.001, 95%), followed by incision location (β = 0.78, *P* < 0.001), and complication rate (β = 0.25, *P* = 0.030). Demographic factors such as gender, age, education level, marital status, and income exhibited notable differences in postoperative cosmetic concerns.

**Conclusion:**

Recurrence risk is the primary driver of surgical preference in PTC patients, followed by cosmetic outcomes. Additionally, postoperative cosmetic concerns varied across different demographic groups. These findings highlight the need for surgeons to consider patient-specific values during preoperative consultations to enhance shared decision-making.

**Clinical Trial Registration:**

https://www.chictr.org.cn/showproj.html?proj=191809, identifier ChiCTR2300069048.

## 1 Background

Papillary thyroid carcinoma (PTC) is a common type of thyroid carcinoma, and surgical treatment has been its main treatment modality (1, 2). However, the conventional open thyroidectomy leaves obvious neck scars, which affects patients’ postoperative appearance and quality of life (3). With the continuous development of minimally invasive surgical techniques, a variety of laparoscopic thyroid surgery methods have emerged, among which transoral endoscopic thyroidectomy vestibular approach (TOETVA) has attracted much attention for its compliance with the principles of natural cavity endoscopic surgery (4–6).

However, with the introduction of these new techniques, a thorough and detailed discussion with the patient becomes especially critical. Such discussions help us assess the relative benefits and risks of each surgical approach. When selecting the appropriate thyroidectomy modality for a patient, a more detailed understanding of the patient’s individual preferences for different surgical approaches can help to optimize communication between doctor and patient. This in turn provides more accurate medical advice to patients and promotes more effective shared patient-doctor decision-making.

A discrete choice experiment (DCE) is a quantitative research method used to assess an individual’s preference for different options (e.g., medical treatment regimens) (7). In a DCE, participants are asked to choose between treatment options with different characteristics and levels to reveal the relative importance they place on these characteristics (8, 9). By simulating real decision-making situations, DCE provides a way to understand people’s decision-making processes, offering researchers the means to delve into individual preferences and weigh different factors. In the medical field, DCE can be used to understand patients’ preferences for different medical interventions, providing important insights into medical decision-making (10–12).

This study investigates the factors influencing PTC patients’ choices between conventional open thyroidectomy and TOETVA using a DCE. While existing literature has explored various aspects of surgical decision-making (13–15), studies employing DCE methodology in this context remain scarce (12). This gap is particularly evident in China, where no prior studies have utilized DCE to comprehensively evaluate patients’ preferences for these two surgical options. Our study aims to fill this gap by providing a deeper understanding of patient preferences, thereby supporting shared decision-making between patients and physicians. The application of an orthogonal design method to generate the questionnaire further enhances the reliability and applicability of the findings, enabling a more patient-centered approach to surgical decision-making.

## 2 Patients and methods

### 2.1 Study setting and participants

This study recruited patients with PTC who were scheduled to undergo thyroidectomy at the thyroid surgery department of Hunan Cancer Hospital, located in Changsha, Hunan Province, China, from March 2023 to July 2023.

Inclusion criteria: (1) aged 18 years or older; (2) diagnosis of PTC by preoperative fine-needle aspiration or intraoperative pathology confirmation, and intended to undergo thyroidectomy; (3) maximum diameter of the tumor was less than 4.0 cm; without parathyroid-related diseases; (4) no obvious contraindications to surgery were seen in routine preoperative examinations; (5) patients voluntarily participated in the study and signed an informed consent form. Exclusion criteria: (1) patients with previous history of thyroid surgery, thyroid isotope therapy, or neck radiotherapy; (2) patients with neurological disease, psychiatric disease, or cognitive impairment who could not correctly describe their own feelings; (3) patients with hyperthyroidism or hypothyroidism; (4) patients with lateral neck lymph node metastasis or distant metastasis; (5) extra-glandular invasion of the tumor; (6) in addition to the above, in the judgment of the investigator as an unsuitable subject for participation. (7) Patients who refused to participate in this study.

In this study, patients first underwent a comprehensive evaluation by the multi-disciplinary treatment (MDT) team of thyroid surgery at Hunan Cancer Hospital to determine their eligibility for inclusion. The MDT’s evaluation included a thorough examination of the patient’s overall health status and collection of medical history. During the evaluation process, patients were provided with a detailed surgical consultation, which included an in-depth analysis of the disease progression, an assessment of the indications for surgery, and a detailed explanation of the two treatment options. focusing on their respective benefits and risks. Eligible participants were then invited to sign a written informed consent form, during which they were given the opportunity to ask questions and had sufficient time for consideration. Upon completion of the signing, participants were asked to complete a face-to-face DCE survey with the study coordinator to understand their preferences for different treatment options.

The study was approved by the Medical Ethics Committee of Hunan Cancer Hospital (2023 NO. 6). Written informed consent was obtained from all individual patients included in this study. In addition, this study was registered with the Chinese Clinical Trial Registry (UIN: ChiCTR2300069048, https://www.chictr.org.cn) in accordance with the World Medical Association’s Declaration of Helsinki, 2013. This study has been reported in line with the STROCSS Guideline (16).

### 2.2 Development of attributes and levels

Participants made choices in a series of hypothetical scenarios featuring different combinations of attributes, which were used to analyze and understand their preferences. The identification of attributes for this DCE was based on a review of existing studies (7, 8, 10, 12, 17–21). The literature review identified several key domains relevant to thyroid surgery decision-making, including oncologic safety, cosmetic outcomes, perioperative burden, and financial cost. These domains were further explored through qualitative interviews with a panel of 8 thyroid surgery patients, selected to ensure diversity in age, gender, educational background, marital status, residential location, and monthly income, as well as 7 expert surgeons with senior academic titles (e.g., Professor or Associate Professor) and extensive clinical experience in thyroid surgery. In these interviews, patients consistently emphasized recurrence risk, visible scarring, and treatment cost as the most salient concerns influencing surgical decision-making. In contrast, surgeons highlighted operative duration and complication risk as clinically important considerations that should also be represented in the DCE. The final list of attributes was subsequently refined through an additional focus group of 8 thyroid surgery patients. Based on these findings, six surgical preference attributes were selected (**Table 1**): incision location (internal/external), incision size (3/5 cm), recurrence rate (1%, 4%, 7%, and 10%), operative duration (75/110 min), complication rate (7%, 9%, 11%, and 13%), and cost (¥15,000/20,000/25,000/30,000).

**Table 1:** Selected attributes and attribute levels.

Attributes	Levels
Incision Location	Internal
	External
Incision Size (cm)	3
	5
Recurrence Rate (%)	1
	4
	7
	10
Surgical Duration (min)	75
	110
Complication Rate (%)	7
	9
	11
	13
Cost (RMB)	¥15,000
	¥20,000
	¥25,000
	¥30,000

Complication rate referred to the overall risk of postoperative surgery-related complications, including common thyroidectomy-related complications and approach-related sensory discomfort.

The selection of attribute levels was guided by clinical relevance, published literature, and expert consensus to ensure that all values were both realistic and interpretable for respondents. Specifically, the recurrence rate levels were designed to represent clinically plausible hypothetical risk scenarios for low-risk PTC rather than precise individualized recurrence estimates. The lowest level of 1% reflected the very low structural recurrence risk reported in ATA low-risk patients, while 10% was used as the upper boundary of a low-risk recurrence scenario. The intermediate values of 4% and 7% were selected to provide evenly spaced and easily interpretable risk differences for respondents. The complication rate referred to the overall risk of postoperative surgery-related complications rather than a single specific complication. It included common thyroidectomy-related complications, such as recurrent laryngeal nerve injury, hypoparathyroidism, postoperative bleeding or infection, as well as approach-related sensory discomfort. For TOETVA, potential approach-specific symptoms, including postoperative numbness or paresthesia in the submandibular, chin, or anterior neck region, were explained to participants during the attribute explanation process. The levels of 7%, 9%, 11%, and 13% were selected to reflect a plausible and clinically meaningful range observed in practice while maintaining sufficient differentiation for respondents. Other attributes, such as incision size and operative duration, were defined based on typical differences between conventional open thyroidectomy and minimally invasive approaches.

A full factorial design would yield 512 (4³ × 2³) possible scenarios. To reduce respondent burden, an orthogonal fractional factorial design was used to generate 16 choice sets (22). The design was generated using a standard orthogonal array approach implemented in statistical software, ensuring attribute level balance and minimal correlation between attributes. Each choice set consisted of two hypothetical surgical alternatives, and all 16 choice tasks were presented to each respondent without blocking into multiple versions. The number of choice tasks (n = 16) was determined based on established DCE methodological recommendations, which suggest that 8–16 tasks per respondent provide an appropriate balance between statistical efficiency and respondent burden.

To improve comprehension, participants were first provided with detailed explanations of all attributes and levels, followed by a warm-up task. In this task, one option contained uniformly favorable attribute levels, while the alternative contained less favorable levels, helping participants understand the decision-making process. A reversed dominance-based consistency check (Question 17) was included to assess response reliability. In this question, the superior option was presented under the opposite label to detect respondents who consistently selected the same option without considering attribute levels. Question 17 was used only for consistency assessment and was not included in the final analysis.

### 2.3 Statistical analysis

The study used SPSS 25.0 to create the database, and dual data entry was used. To assess the normality of the data distribution, we performed the Shapiro-Wilk test for normality. If the data were normally distributed, descriptive statistics were reported as mean ± standard deviation; otherwise, the median and interquartile range (IQR) were used to describe the continuous data. Dichotomous data were described using frequencies and proportions. Comparisons between groups were made using chi-square tests or t-tests, depending on the distribution of the data. Data from the DCE component were analyzed using a mixed logit model for regression analysis, with the help of latent category analysis for preference heterogeneity analysis. Each survey had 16 choice tasks consisting of 2 scenarios, and each respondent completed 16 choice tasks. Results are reported as coefficients with 95% confidence intervals (CIs). The sign of the coefficient indicates the direction of the attribute’s influence on individual preferences. The larger the absolute value of the coefficient, the stronger the influence of the attribute on the individual’s choice.

In this study, surgical choice was used as the dependent variable, and the six surgical-related attributes included were used as independent variables for regression analysis using a mixed logit model, mainly implemented using the mixlogit command in Stata 15.0. Among them, cost was used as a continuous variable, and the other five variables were treated as dummy variables to be introduced into the model analysis.

Willingness to pay (WTP) denotes a measure of the monetary value of each referral attribute by the study population.


WTP patients =(β patients/β cost)*(-1000)

## 3 Results

### 3.1 Participants

**Figure 1:**
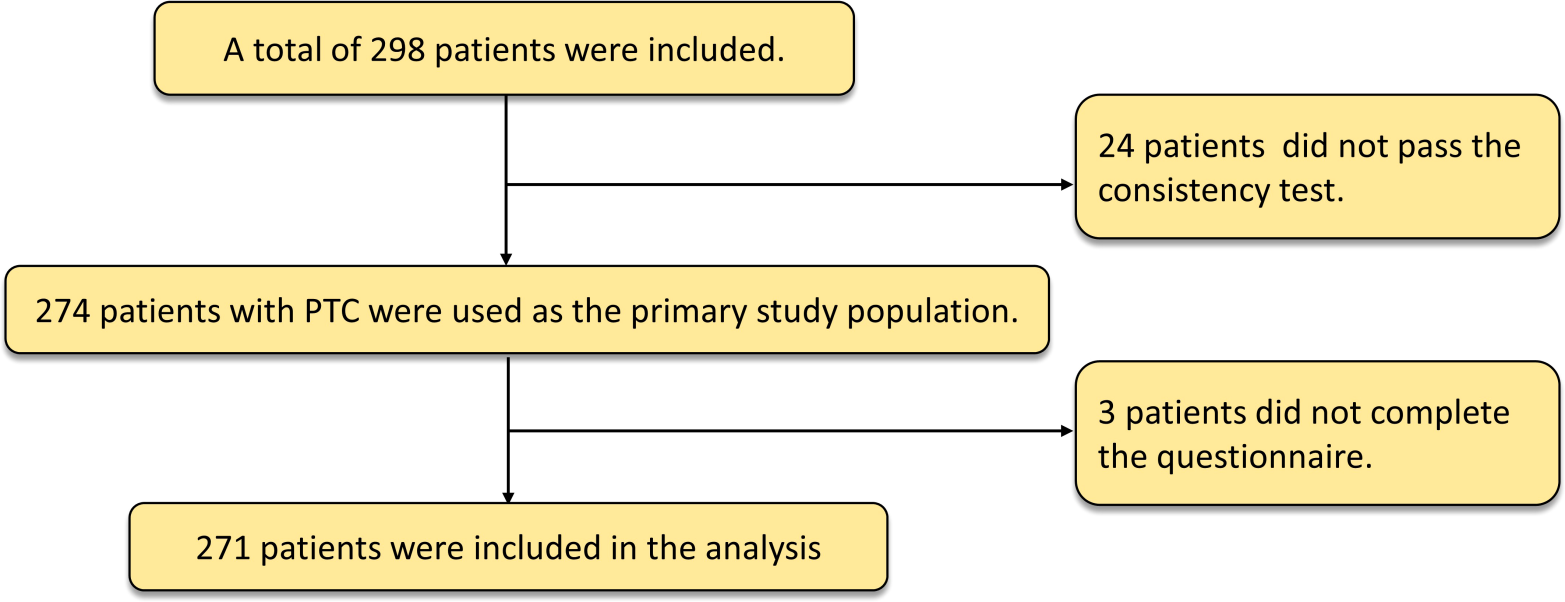
Flow diagram shows the process of study selection.

Based on previous studies (17, 23), a total of 298 participants were included in this study. Three patients who did not complete the DCE questionnaire and 24 respondents who failed the consistency test were excluded, resulting in 271 patients with PTC as the main subjects for the DCE (**Figure 1**). Among the 271 participants, 214 (78.97%) underwent TOETVA, and the remaining 57 (21.03%) underwent conventional open thyroidectomy. Of these, 49 (18.1%) were male and 222 (81.9%) were female, with a median age of 36.0 years (IQR: 30.5–46.0 years). In terms of educational attainment, 63.5% had higher education, and 36.5% had high school education or below. In terms of marital status, 74.5% were married, with the majority of the remaining being unmarried (20.3%). The participants predominantly lived in urban areas (62.7%), with rural residents making up 37.4%. Income distribution was relatively even, but the lower-middle income group was the largest, with 27.3% of the participants earning between ¥3,000 and ¥3,999 per month (approximately $420 to $560 USD, using an exchange rate of ¥7 to $1 USD). Household incomes were mainly between ¥6,000 to ¥9,999 and ¥10,000 to ¥19,999. The vast majority of participants (53.9%) had medicare, with a significant proportion also covered by Medicaid (**Table 2**).

**Table 2:** Respondent characteristics (n=271).

Characteristics	Groups	Frequency (n, %)	
Age (years)	≤45	201	74.2
	>45	70	25.8
Sex	Male	49	18.1
	Female	222	81.9
Education	Elementary school and below	20	7.4
	Junior high school	39	14.4
	Senior high school	40	14.7
	University/College	159	58.7
	Graduate	13	4.8
Personal status	Single	55	20.3
	Married	202	74.5
	Divorced	10	3.7
	Widowed	4	1.5
Residential Area	Urban	170	62.7
	Rural	101	37.3
Individual monthly income	0-1,499	54	19.9
	1,500-2,999	47	17.3
	3,000-3,999	74	27.3
	5,000-7,999	47	17.3
	8,000-9,999	27	10.0
	10,000-19,999	15	5.6
	≥20,000	7	2.6
Household monthly income	0-2,999	17	6.3
	3,000-5,999	41	15.1
	6,000-9,999	73	26.9
	10,000-19,999	61	22.5
	20,000-49,999	27	10.0
	50,000-99,999	28	10.3
	≥100,000	24	8.9
Insurance	Medicare	146	53.9
	Medicaid	118	43.5
	Self-pay	3	1.1
	Commercial insurance	4	1.5

### 3.2 Preferences and willingness-to-pay

The regression results showed that, except for the duration of surgery, all six attributes included in this study had a significant effect on the choice of surgical regimen, and the direction of the regression coefficients was generally consistent with expectations. When faced with a choice of surgical procedure, participants preferred options with lower cost, lower recurrence rate, internal incision location, smaller incision size, and lower complication rate. Among all the non-economic factors, the recurrence rate was the most concerned factor for PTC patients (β = 8.18, *P* < 0.001, 95% CI 7.24 to 9.12), followed by the location of incision (β = 0.78, *P* < 0.001, 95% CI 0.51 to 1.05), the incidence of complications (β = 0.25, *P* = 0.030, 95% CI 0.02 to 0.47), and the size of incision (β = 0.21, *P* = 0.004, 95% CI 0.07 to 0.36) (**Table 3**).

**Table 3:** Estimated coefficients and marginal willingness to pay.

Attributes	Levels	Coefficient in analysis (β)	*P*-value	95% CI		Willingness-to-pay (¥)
Cost (¥)		-.041	<0.001	-0.06	-0.02	
Incision Location	Inside	0.78	<0.001	0.51	1.05	19141.14
	Outside					
Incision Size (cm)	3	0.21	0.004	0.07	0.36	5285.90
	5					
Recurrence Rate (%)	1	8.18	<0.001	7.24	9.12	201409.60
	4	4.50	<0.001	4.10	4.90	110771.70
	7	2.17	<0.001	1.95	2.40	53439.20
	10					
Surgical Duration (min)	75	0.11	0.122	-0.03	0.25	2661.38
	110					
Complication Rate (%)	7	0.25	0.030	0.02	0.47	6038.97
	9	0.15	0.140	-0.05	0.36	3782.01
	11	0.34	0.015	0.07	0.62	8473.33
	13					

Based on the regression results of the mixed logit model, it can be concluded that participants’ valuation of the monetary value of each surgical attribute level, the most important non-economic factor influencing surgical options is the recurrence rate. Participants are more willing to spend an additional ¥53,439.20 (*P* < 0.001) to choose an option with a recurrence rate of 7% compared to a recurrence rate of 10%, ¥110,771.70 (*P* < 0.001) for a recurrence rate of 4%, and even ¥201,409.60 (*P* < 0.001) to reduce the recurrence rate to 1%. Similarly, the incision location had a significant impact on participants’ choices, as they were willing to spend an additional ¥19,141.14 (*P* < 0.001) when the surgical incision was changed from external to internal. Similarly, participants were also willing to spend an additional ¥5,285.90 (*P* = 0.004) to reduce a 5 cm incision to 3 cm. The complication rate also had an impact on surgical options, although patients preferred lower postoperative complication rates, the willingness-to-pay results showed no significant trend in patient preference between lower levels of recurrence rates, with patients willing to spend an additional ¥8,473.33 (*P* = 0.015) or ¥6,038.97 (*P* = 0.030) to reduce complications to 11% or 7%, respectively, compared to a 13% recurrence rate (**Table 3**).

### 3.3 Heterogeneity of preferences

In order to explore the cosmetic concerns of different groups regarding thyroid surgery, we conducted stratified statistical analysis based on demographic characteristics and arrived at the following conclusions (**Table 4**).

**Table 4:** Estimated coefficients and marginal willingness to pay after stratification of groups.

Characteristics	Groups (n, %)	Attributes	Coefficient (β)	*P* value	Willingness-to-pay (¥)
Age	≤30 (68, 25.1)	Cost	-0.03	0.086	–
		Inside	1.45	0.001	42343.09
	>30 (203, 74.9)	Cost	-0.05	<0.001	–
		Inside	0.75	<0.001	16647.99
	≤40 (165, 60.9)	Cost	-0.06	<0.001	–
		Inside	1.10	<0.001	23592.51
	>40 (106, 39.1)	Cost	-0.04	0.003	–
		Inside	0.49	0.008	13996.48
	≤50 (221, 81.6)	Cost	-0.04	<0.001	–
		Inside	0.90	<0.001	21889.39
	>50 (50, 18.4)	Cost	-0.05	0.005	–
		Inside	0.61	0.018	12997.91
Sex	Female (49, 18.1)	Cost	-0.05	<0.001	
		Inside	0.91	<0.001	19631.47
	Male (222, 81.9)	Cost	-0.02	0.32	
		Inside	0.34	0.24	
Education	High school and less than high school (99 36.5)	Cost	-0.03	0.002	
		Inside	0.34	0.022	9704.46
	More than high school (172, 63.5)	Cost	-0.05	<0.001	
		Inside	1.46	<0.001	27042.02
Personal status	Unaccompanied (69, 25.5)	Cost	-0.05	<0.001	
		Inside	0.67	<0.001	14859.56
	be married (202, 74.5)	Cost	-0.03	0.158	
		Inside	1.17	<0.001	45396.14
Residential Area	Rural (170, 62.7)	Cost	-0.03	0.016	
		Inside	0.17	0.245	
	Urban (101, 37.3)	Cost	-0.05	<0.001	
		Inside	1.36	<0.001	26943.48
		Inside	0.61	0.018	12997.91
Individual monthly income	≤2999 (101, 37.3)	Cost	-0.03	0.015	
		Inside	0.38	0.036	13917.88
	>2999 (170, 62.7)	Cost	-0.06	<0.001	
		Inside	1.24	<0.001	22164.44
Household monthly income	≤9999 (131, 48.3)	Cost	-0.04	0.001	
		Inside	0.47	0.004	12695.62
	>9999 (140, 51.7)	Cost	-0.05	0.001	
		Inside	1.30	<0.001	28010.56
Insurance	High reimbursement (150, 55.4)	Cost	-0.04	0.014	
		Inside	0.93	<0.001	20905.49
	Low reimbursement (121, 44.6)	Cost	-0.04	<0.001	
		Inside	0.75	<0.001	18149.88

1. Age is an important factor affecting the postoperative cosmetic attention of patients. Younger patients were more concerned about postoperative cosmetic outcomes and were willing to pay more to avoid postoperative scars. Specifically, with participants aged ≤30, ≤40, and ≤50 willing to pay additional ¥42343.09 (*P* = 0.001), ¥23592.51(*P* < 0.001) and ¥21889.39(*P* < 0.001) for a scarless procedure, respectively. In contrast, patients older than 30, 40 and 50 years were only willing to pay an additional ¥16647.99(*P* < 0.001), ¥13996.48(*P* = 0.008) and ¥12997.91(*P* = 0.018), respectively.
2. Female patients were more concerned about the cosmetic outcome of the surgery than male patients, with the former willing to pay an additional ¥19,631.47(*P* < 0.001) for a scarless surgical option. In contrast, male patients were not particularly concerned about the presence of scars post-surgery.
3. In terms of education, patients with higher education were willing to pay an additional ¥27,042.02(*P* < 0.001) for a scarless procedure, whereas those with secondary or lower education were only willing to pay an additional ¥9,704.46(*P* = 0.022).
4. Regarding marital status, the married patients did not care about the cost of surgery (*P* = 0.158) and were willing to pay an additional ¥45,396.14(*P* < 0.001) for a scarless procedure, while Patients without a partner (including single, divorced and widowed) will consider the cost when choosing the operation method (*P* < 0.001) and were only willing to pay an additional ¥14,859.56(*P* < 0.001) to avoid neck scars.
5. Geographically, urban residents were willing to pay an extra ¥26,943.48(*P* < 0.001) for a scarless procedure, while rural residents were not particularly concerned about post-surgery scars (*P* = 0.245).
6. In terms of income, participants with a personal monthly income higher than ¥2,999 and those with a family monthly income higher than ¥9,999 were willing to pay an additional ¥22,164.44(*P* < 0.001) and ¥28,010.56(*P* < 0.001), respectively, for TOETVA option, while patients with lower personal and family incomes were only willing to pay an additional ¥13,917.88(*P* = 0.036) and ¥12,695.62(*P* = 0.004), respectively.
7. In terms of insurance, participants with high reimbursement insurance were willing to pay an additional ¥20905.49(*P* < 0.001) for a scarless procedure, while those with low reimbursement insurance were only willing to pay an additional ¥18149.88(*P* < 0.001).

## 4 Discussion

In this study, we endeavored to gain insight into patient preferences and considerations in thyroid surgery modality selection. Patient concerns regarding surgical choices are multifaceted and broad, encompassing various aspects such as long-term surgical outcomes, postoperative aesthetics, and financial considerations. We particularly focused on the importance that different populations place on postoperative scarring. Through this study, we aim to provide physicians with deeper insights to better understand patient expectations, enhance the personalization of surgical decisions, and improve patient satisfaction.

In terms of the economic attribute, specifically cost, patients showed a clear preference for surgical options with lower costs. This finding is consistent with previous research that underscores the importance of cost in patients’ decision-making processes (24, 25). Such decisions are particularly influenced by concerns over the financial strain imposed by surgery costs, which may be compounded by the broader context of escalating healthcare expenses (26). The negative preference towards higher-cost surgeries, as observed in this study, reflects a broader trend in patient behavior where cost becomes a major determinant in treatment selection. This is especially relevant in healthcare systems where financial accessibility is a significant factor (27, 28). Acknowledging and addressing the economic barriers faced by patients can lead to more informed and satisfying treatment choices (29). Therefore, physicians should consider the financial concerns of patients when providing surgical recommendations. By offering more affordable alternatives or discussing cost-effective treatment plans, doctors can help alleviate financial burdens and improve patient satisfaction, ultimately leading to better overall outcomes. In addition to cost considerations, patients expressed significant concern about the risk of postoperative recurrence, preferring surgical options with lower recurrence rates, highlighting their deep sensitivity to the long-term outcomes of surgery. This may reflect patients’ concerns about their health status and the potential physical and psychological burden of cancer recurrence. However, a North American study (18) found that 20% of non-thyroid cancer patients surveyed preferred transaxillary endoscopic thyroidectomy without neck scarring, even though it did not cure their disease. This preference may be partially attributed to the fact that the subjects in the North American study were predominantly non-thyroid disease patients and were unable to empathize with the sentiments of patients with a confirmed PTC diagnosis. This observation is supported by other studies, which have shown that when patients know that their test results are cancerous or possibly cancerous, they are more likely to opt for more aggressive surgical treatments rather than choosing to remain under observation (7, 19). Similarly, a study in the United States has shown that patients diagnosed with thyroid cancer are more concerned with surgeon experience and the safety and thoroughness of surgery than with surgical scarring (20).

In addition, we found that the presence or absence of a scar, as well as the length of the scar, played an equally crucial role in patients’ decision-making. This is consistent with other findings, as several surveys in North America have shown that patients preferred neck scarless transoral vestibular approach or transaxillary approach thyroidectomy over conventional open thyroidectomy, even if it was more costly and took longer to perform (18, 20). Additionally, patients prefer shorter and thinner incisions if neck scars are present (21).

This highlights the universal concern of patients worldwide about aesthetics, especially regarding scarring (30), which is also evident in thyroid surgery decision-making (21, 31, 32). The surgical scar from conventional open thyroidectomy is located on the neck, making it difficult to conceal with clothing and often causing psychological discomfort and social stigmatization in patients (33). The role of cosmetic concerns in thyroid surgery decisions is underscored by a recent study that used eye-tracking software. The study found that the presence of visible neck scarring diverts viewers’ attention from the face to the neck, a distraction not experienced by patients undergoing TOETVA (34). This transcends cultural and geographic differences, impacting patients’ quality of life and psychological well-being universally. Considering patients’ concerns about surgical scars, surgeons should fully understand patients’ aesthetic preferences and sensitivity to appearance during preoperative consultations. Personalized preoperative communication can help patients better understand potential scarring after surgery and provide optional surgical approaches to meet patient expectations and improve satisfaction with surgical decision-making (35, 36). Additionally, surgeons can introduce postoperative scar management methods to alleviate scar anxiety and promote better postoperative recovery and psychological well-being (37, 38).

In terms of postoperative complications, we have observed some interesting patterns. Studies indicate that patients in North America are particularly concerned about injuries to the laryngeal recurrent and chin nerves during surgery (12). However, our study revealed fluctuating levels of concern among patients regarding postoperative complications. While patients generally prefer lower rates of postoperative complications, our findings show that there was no significant preference among patients for lower rates of recurrence.

These findings demonstrate a clear contrast between our study and previous studies conducted in North America. North American studies have often involved individuals with benign thyroid disease or members of the general public, in whom concerns about recurrence are either absent or largely hypothetical. In these settings, patients tend to prioritize cosmetic outcomes and the risk of surgical complications, and may even accept higher risks or costs in exchange for avoiding visible scarring.

In contrast, our study specifically enrolled patients with confirmed papillary thyroid carcinoma (PTC), in whom recurrence represents a real and immediate concern. Accordingly, recurrence risk emerged as the most important factor influencing surgical decision-making, outweighing both cosmetic concerns and complication risk. This contrast reflects a fundamental difference in patient priorities. In non-cancer populations, preferences are largely driven by aesthetic and perioperative considerations, whereas in patients with confirmed malignancy, decision-making is primarily guided by concerns about long-term oncologic outcomes. This finding highlights the critical role of disease context in shaping patient preferences and suggests that results from non-cancer populations may not be directly generalizable to patients with malignant disease.

Interestingly, operative duration did not significantly influence patients’ surgical choices in this study. Several possible explanations may account for this finding. First, in the context of cancer surgery, patients may prioritize attributes directly related to long-term prognosis, such as recurrence risk, over relatively short differences in operative time. A difference of several tens of minutes may be perceived as clinically less meaningful by patients, particularly when surgery is performed under general anesthesia and the duration is largely outside the patient’s direct experience. Second, operative time may be regarded by patients as a technical factor mainly determined by the surgeon and surgical team, rather than as an attribute that directly affects postoperative recovery or quality of life. Third, the levels used in our DCE (75 and 110 minutes) may not have been sufficiently different to generate a strong preference signal. Therefore, the non-significant effect of operative duration should be interpreted in the context of both patient perception and the specific attribute levels used in this study.

To gain insight into the manifestation of cosmetic concerns in thyroid surgery among different populations, we conducted an in-depth stratified analysis. This analysis took into account factors such as gender, education level, presence of a partner, place of residence, personal financial status, and insurance purchasing method. The aim of this analysis was to explore the unique concerns of different populations regarding postoperative scarring when faced with surgical decision-making. Through this stratified study, we aim to provide physicians with a more comprehensive understanding to more accurately respond to their patients’ cosmetic needs.

When stratifying patients by age, our study revealed significant differences in the emphasis placed on postoperative appearance across different age groups of patients. While all age groups showed a concern for appearance, younger patients demonstrated a stronger desire for scarless outcomes. Specifically, we observed that patients aged 30 and below were willing to pay an additional ¥42,343.09 for TOETVA, whereas this willingness gradually decreased with age, with patients over 50 willing to pay only ¥12,997.91 for TOETVA. This aligns with other research indicating that younger patients prefer TOETVA and are willing to incur additional costs for it (12, 18). With the increasing trend of thyroid cancer affecting younger individuals (39), our study emphasizes the need for physicians to prioritize discussions on appearance concerns during preoperative consultations with younger patients. As younger patients are more likely to prioritize cosmetic outcomes, preoperative consultations tailored to their needs should highlight the potential benefits of scarless surgery and ensure they are fully informed about the available surgical options that align with their aesthetic expectations and values.

In terms of gender, we found that female patients were more concerned about the cosmetic outcome of the surgery compared to males. They were willing to pay an additional ¥19,631.47 to opt for a scar-free procedure, whereas male patients were not very concerned about the presence of postoperative scars. The reasons why women are more concerned about postoperative scarring in their thyroid surgery decision-making are multidimensional, including sociocultural factors, aesthetic concepts, and specific health considerations. In East Asian cultures, women often face greater aesthetic pressure (40), and factors that affect appearance, such as visible postoperative scars on the face or neck, may have a more significant psychological and social impact for them. Given this, it is important for doctors to be more attuned to the aesthetic needs of female patients, providing targeted information to help them make decisions that align with both medical requirements and personal aesthetic expectations. While such concerns may also affect male patients, the impact tends to be more pronounced for women (41, 42).

Our study also identified marital status, education level, place of residence, personal financial status, and insurance as important factors influencing patients’ cosmetic concerns. Patients with a partner demonstrated a greater willingness to pay for scarless surgery, which may be related to the influence of close interpersonal relationships on self-image and appearance expectations, as well as the role of joint household decision-making and shared financial resources. Similarly, patients with higher education levels were more willing to pay to avoid visible scars, possibly reflecting greater health literacy, stronger awareness of quality-of-life outcomes, and a higher valuation of long-term psychosocial well-being. Urban residents and individuals with higher income levels also showed increased willingness to pay, which may be attributed to greater financial capacity and heightened sensitivity to appearance-related social and professional factors. In addition, differences across insurance types may reflect variations in perceived financial burden and reimbursement expectations, influencing how patients weigh cost against aesthetic benefits. These findings highlight the importance of considering socioeconomic and interpersonal factors during preoperative consultation and support the need for more individualized communication strategies to better align surgical decision-making with patient values and expectations.

The preference weights identified in this study may provide a practical framework for improving preoperative shared decision-making in PTC surgery. Our findings suggest that clinicians should prioritize clear and accurate communication regarding recurrence risk and oncologic safety, as these factors carried the greatest weight in surgical decision-making. Information about cosmetic outcomes, incision location, and scar visibility should then be discussed in relation to each patient’s individual values and demographic characteristics. For example, younger patients, female patients, and patients with higher education or income levels may require more detailed counseling regarding cosmetic outcomes and scar-related concerns. However, the cosmetic advantages of minimally invasive techniques should be presented in a balanced manner and should not overshadow discussions of oncologic safety, complication risk, and treatment cost. In the future, DCE-derived preference weights could be incorporated into structured decision aids or consultation checklists to help patients compare surgical options more transparently and to support preference-sensitive surgical decision-making.

There are some limitations to the study, firstly, the source of subjects was limited to one hospital and mainly from a single province, which may limit the external validity of the findings as it may not be fully representative of the diversity of patients undergoing thyroid surgery in our country or even globally.

Second, the final sample was heavily skewed toward patients who ultimately underwent TOETVA rather than conventional open thyroidectomy. This imbalance may introduce selection bias and should be considered when interpreting the findings. Our institution is a national training center for TOETVA in China, and some patients may have specifically sought care at our center because of their interest in minimally invasive or scarless surgical approaches. Therefore, the study population may have been enriched for patients who were relatively more receptive to TOETVA, rather than fully representing the broader population of all patients with PTC. In addition, all participants completed the survey after a preoperative consultation with the surgeon, which may have further influenced both the observed sample distribution and the stated preferences elicited in the DCE. The content, tone, and framing of the clinical consultation, together with social desirability bias and the inherent power dynamics in the surgeon–patient relationship, may have encouraged some patients to provide responses they perceived as more acceptable in the clinical context. As a result, the estimated preference weights, particularly for attributes related to cosmetic benefit and treatment burden, should be interpreted with caution, as they may reflect a combination of intrinsic patient preferences and context-dependent influences.

Finally, standardized risk and cost levels for each method of thyroidectomy were used in the study, which did not take into account individual differences in risk and cost that may result from geographic differences in thyroid pathology, patient comorbidities, surgeon experience, and cost of living. These limitations, including the exploratory nature of the subgroup analyses and the small sample size, should be carefully considered when interpreting the study results and developing relevant policies.

## 5 Conclusions

We found that the most important factor of concern for PTC patients was the recurrence rate after surgery, followed by the presence and size of surgical scars. In addition, factors such as gender, age, marital status, education level, place of residence, personal financial status, and insurance significantly influenced the cosmetic concerns of PTC patients. This finding provides useful information for thyroid surgeons to provide more nuanced preoperative counseling to patients and to meet their individual needs, thus facilitating more informed surgical decisions. However, future multicenter studies with large sample sizes are needed to further clarify the selection preferences of different patients and to improve the external validity and generalizability of the findings.

## Data Availability

The original contributions presented in the study are included in the article/supplementary material. Further inquiries can be directed to the corresponding author.
